# In Vitro Evaluation of Halotolerant *Bacillus velezensis* 24.5 as a Promising Probiotic with Broad-Spectrum Antimicrobial Activity

**DOI:** 10.3390/microorganisms13102240

**Published:** 2025-09-24

**Authors:** Filofteia Camelia Diguță, Radu Cristian Toma, Florentina Matei

**Affiliations:** 1 Faculty of Biotechnologies, University of Agronomic Sciences and Veterinary Medicine, 59 Marasti Blvd., 011464 Bucharest, Romania; camelia.diguta@bth.usamv.ro (F.C.D.); florentina.matei@unitbv.ro (F.M.); 2 Faculty of Food Industry and Tourism, Transilvania University of Brașov, 148 Castelului Street, 500014 Brașov, Romania

**Keywords:** *Bacillus velezensis*, molecular identification, antimicrobial activity, gastrointestinal tolerance, safety

## Abstract

The remarkable metabolic adaptability of *Bacillus velezensis,* including efficient nutrient use, spore formation, and the secretion of antimicrobial peptides, supports its expanding role in biotechnological applications ranging from crop protection to probiotic development. In this study, the halotolerant strain 24.5 was identified as *B. velezensis* through 16S rDNA and *gyrA* gene sequencing. PCR analyses confirmed the presence of genes responsible for polyketides, lipopeptides, and dipeptides biosynthesis. These results indicate the potential for the production of structurally diverse bioactive metabolites. Strain 24.5 demonstrated remarkable antimicrobial activity against 19 bacterial pathogens and three *Candida* species (*p* < 0.05). The study demonstrated high survival rates under simulated gastrointestinal conditions, suggesting strong adaptability for gut colonization. Antioxidant evaluation revealed DPPH radical scavenging activities of 34.68% for intact cells and 18.47% for the cell-free extract (*p* < 0.05). The enzymatic profile highlighted versatile metabolic functions, supporting its multifaceted probiotic potential. Auto-aggregation reached 84.42% at 24 h, and high hydrophobicity toward hexane (71.62%) supported adhesion potential. Antibiotic susceptibility profiling showed sensitivity or intermediate susceptibility to 22 of 24 tested antibiotics (*p* < 0.05). No haemolytic activity was detected, supporting its safety profile. Overall, these results emphasise the adaptability and multifunctional properties of *Bacillus velezensis* strain 24.5, highlighting its potential as a promising probiotic candidate for applications in food safety and biotechnology.

## 1. Introduction

Probiotics are beneficial microorganisms that positively influence host health when administered in adequate amounts [1,2]. The most common probiotic genera include *Lactobacillus* (recently reclassified into several new genera), *Bifidobacterium*, *Enterococcus*, *Lactococcus*, *Streptococcus*, and *Pediococcus* [3,4,5,6,7,8,9], as well as yeasts such as *Saccharomyces boulardii* and *S. cerevisiae* [10,11,12,13]. Their health benefits are mediated by multiple mechanisms, including modulation of the gut microbiota, competitive exclusion of pathogens, enhancement of epithelial barrier integrity, immune modulation, and bioactive metabolite production [3,4,14]. While *Lactobacillus* and *Bifidobacterium* have traditionally dominated the probiotic market [3,4,5,6], their viability can be reduced by environmental stress or preservation techniques [15,16].

In contrast, spore-forming *Bacillus* species exhibit exceptional resilience under harsh conditions, including in the gastrointestinal tract, and produce diverse bioactive metabolites, making them attractive next-generation probiotics [17,18,19,20,21,22,23]. Several *Bacillus* species, including *B. subtilis* and *B. velezensis*, and their postbiotics are recognized as safe by the U.S. Food and Drug Administration (GRAS) and the European Food Safety Authority (QPS) [24,25]. *Bacillus velezensis* is well characterized for its biocontrol capabilities, inhibiting a broad range of bacterial and fungal phytopathogens and supporting plant health [26,27,28,29]. However, its probiotic use requires strain-specific safety assessments, such as genomic and phenotypic analyses, to confirm the absence of virulence factors, enterotoxins, and transferable antibiotic resistance genes [21,30,31,32]. This species produces a wide array of secondary metabolites, including antimicrobial peptides, polyketides, and lipopeptides, which disrupt microbial membranes, inhibit biofilms, and modulate host immune responses [33,34,35,36,37,38]. It also exhibits a diverse enzymatic profile that enhances nutrient digestibility and improves feed conversion efficiency in the host [20,39,40,41]. Strains of *B. velezensis* are considered safe for use in animal feed and aquaculture [31,32,41,42] and are increasingly investigated for probiotic applications in both human and veterinary fields [25,36,38,40]. Due to the strain-dependent nature of probiotic effects, each candidate must undergo thorough testing to ensure both safety and efficacy.

Building on the proven traits of halotolerant strain 24.5, previously identified as a biocontrol agent against phytopathogenic fungi [29], this study thoroughly characterizes it as a next-generation probiotic, emphasizing its versatility and potential to expand biotechnological applications.

## 2. Materials and Methods

### 2.1. Preparation of Bacterial Strains and Growth Conditions

Strain 24.5 from the Microorganisms Collection of the Faculty of Biotechnologies, UASMV Bucharest, preserved in 40% (*v*/*v*) glycerol at −20 °C, was used in this study. The strain was previously isolated from hypersaline water in Lopătari, Romania, by Proca et al. [29], and has been reported to show biocontrol potential against phytopathogenic fungi. Before use, the strain 24.5 was revived twice on tryptic soy agar (TSA; Scharlab S.L., Barcelona, Spain) and incubated at 37 °C for 24 h.

The inhibitory activity of strain 24.5 was determined against a panel of reference microorganisms. The pathogenic group included *Streptococcus pyogenes* ATCC 19615, *Listeria monocytogenes* ATCC 7644, *L. ivanovii* ATCC 19119, *Salmonella enterica serovar* Typhimurium ATCC 14028, *S. enterica serovar* Enteritidis ATCC 13076, *Staphylococcus aureus* ATCC 25923, methicillin-resistant *Staph. aureus* (MRSA) ATCC 33592, *Escherichia coli* ATCC 11229, *E. coli* ATCC 8739, *Pseudomonas aeruginosa* ATCC 15442, *Ps. aeruginosa* ATCC 27853, *Proteus vulgaris* ATCC 13315, and *Serratia marcescens* ATCC 14756.

The commensal or opportunistic group comprised *Bacillus cereus* ATCC 11778, *Staphylococcus epidermidis* ATCC 12228, methicillin-resistant *Staph. epidermidis* (MRSE) ATCC 51625, *Enterococcus faecium* ATCC 6057, *Ent. faecalis* ATCC 29212, *Ent. hirae* ATCC 10541, *Listeria innocua* ATCC 33090, and *Rhodococcus equi* ATCC 8939.

Additionally, four *Candida* strains were included in the assay: *C. albicans* ATCC 10231, *C. glabrata* ATCC 2001, *C. parapsilosis* ATCC 20019, and *C. tropicalis* ATCC 44508.

Bacterial strains were routinely maintained on TSA, while *Candida* strains were maintained on potato dextrose agar (PDA; Alliance Bio Expertise, Guipry-Messac, France).

### 2.2. Molecular Characterization of 24.5

#### 2.2.1. Molecular Identification of Strain 24.5

Genomic DNA extraction was performed using the Quick-DNA™ Fungal/Bacterial Miniprep Kit (Zymo Research, Irvine, CA, USA) according to the manufacturer’s guidelines. The taxonomic identity of strain 24.5 was confirmed through amplification and sequencing of the 16S rRNA and *gyrA* genes, following the primers and protocols described by Boiu-Sicuia et al. [28] and Chun and Bae [43]. PCR products were sequenced by Cellular and Molecular Immunological Application (Larissa, Greece). The resulting sequences were compared against the National Center for Biotechnology Information (NCBI) database using the Basic Local Alignment Search Tool (BLAST; https://blast.ncbi.nlm.nih.gov/Blast.cgi, accessed on 25 January 2025) to assess sequence homology. Phylogenetic relationships were inferred in MEGA X [44] using the neighbor-joining method based on DNA distance algorithms [45].

#### 2.2.2. Detection of Bioactive Metabolite-Encoding Genes

Genes linked to bioactive metabolites production were identified using singleplex PCR. Primer sequences for each target gene are listed in Table 1. Singleplex PCR reactions (25 µL) included 1× DreamTaq™ Green Buffer (20 mM MgCl_2_; Thermo Fisher Scientific, Baltics, UAB, Vilnius, Lithuania), 0.5 µM of each primer, 0.2 mM dNTPs, 10 ng of genomic DNA, and 0.025 U DreamTaq™ DNA Polymerase, with the final volume adjusted with DNase-free ultrapure water. Reactions were run on a Multi-Gene™ Thermal Cycler (Labnet International, Inc., Cambridge, UK). PCR products were separated on 2% (*w*/*v*) agarose gels (VWR International BVBA, Leuven, Belgium) at 90 V for 60 min, visualized under UV light, and compared with a molecular weight marker (100 bp DNA Ladder Ready-to-Load, Solis BioDyne OÜ, Tartu, Estonia).

### 2.3. Functional Properties of 24.5 Strains

#### 2.3.1. Antimicrobial Activity

The spot diffusion technique was employed to evaluate the antimicrobial activity of strain 24.5, following the method described by Toma et al. [50]. All target strains were freshly subcultured and incubated at 37 °C for 24 h. Indicator cultures were adjusted to a 0.5 McFarland standard in sterile saline solution (0.9%NaCl) and uniformly spread onto fresh TSA for bacterial strains or PDA for yeast strains, using sterile cotton swabs. A 5 µL aliquot of an overnight culture of strain 24.5, grown in tryptic soy broth (TSB; Scharlab S.L., Barcelona, Spain) at 37 °C, was spotted onto the inoculated agar surfaces. Plates were left to dry under sterile conditions for approximately 15 min before incubation at 37 °C for 24–48 h, depending on the growth rate of the indicator organism. Antimicrobial activity was quantified as the diameter of the inhibition zone, measured in millimeters, and excluding the colony spot.

#### 2.3.2. Gastrointestinal Tolerance Assay

The tolerance of *B. velezensis* strain 24.5 to simulated gastrointestinal conditions was assessed using a modified protocol from da Rosa et al. [32] and Borah et al. [51]. An overnight culture in TSB incubated at 37 °C and 150 rpm was harvested by centrifugation (2000× *g*, 10 min), washed twice with phosphate-buffered saline (PBS; pH 7.2; VWR International, Rosny-sous-Bois, France), and adjusted to approximately 10^9^ CFU/mL. For gastric tolerance testing, 1 mL of the standardized suspension was inoculated into simulated gastric fluid (PBS adjusted to pH 2.0 with 0.1 M HCl and supplemented with 0.3% *w*/*v* pepsin) and incubated at 37 °C for 3 h. Bile salt tolerance was evaluated by inoculating the strain 24.5 into TSB adjusted to pH 8.0 and supplemented with bile salt at final concentrations of 0.3–2% (*w*/*v*). Unmodified TSB served as the control. Cultures were incubated at 37 °C for 4 h, after which samples were collected for viable counts. The survival rate of strain 24.5, expressed as a percentage, was obtained using the equation provided below:

$$SR\;(\%)\;=\;\frac{log\;CFUtSGC\;or\;SIC}{log\;CFUi}\;\times \;100$$

where *CFUi* represents the initial colony-forming units, and *CFUt* denotes the viable counts at the respective time intervals under simulated gastric conditions (SGC) or simulated intestinal conditions (SIC) at defined time points.

#### 2.3.3. Antioxidant Effect

The antioxidant capacity of strain 24.5 was assessed using the DPPH radical-scavenging assay, following the procedure of Coulibaly et al. [52] with minor modifications. An overnight culture was centrifuged at 4000× *g* for 10 min, and the resulting cell pellet was washed twice with PBS (pH 7.2). The suspension was adjusted to approximately 10^9^ CFU/mL in PBS. For preparation of intracellular cell-free extracts, the washed cells were disrupted in a bead beater for three cycles of 30 s agitation followed by 10 s rest. For the assay, 1 mL of either the intact cell suspension or the cell-free extract was mixed with 2 mL of freshly prepared DPPH solution (100 µM in methanol; Alfa Aesar, Kandel, Germany) and incubated in the dark at room temperature for 30 min. The samples were clarified by centrifugation at 6000× *g* for 2 min before reading the absorbance at 517 nm using a spectrophotometer (UV-1800 spectrophotometer, ChromTech, Minneapolis, MN, USA). The deionized water was the negative control. The transition in color from purple to yellow was interpreted as an indication of radical scavenging. The scavenging activity (%) was determined according to the equation:

$$Antioxidant\;activity\;(\%)=(1-\frac{Asample}{ADPPH})\times 100.$$

#### 2.3.4. Enzymatic Activity Assays

Strain 24.5 was grown on selective agar plates and incubated at 37 °C for 72 h. Extracellular hydrolytic activities were assessed on TSA containing specific substrates: 1% soluble starch for amylase, 1% carboxymethyl cellulose (CMC) for cellulase, 1% Tween 80 with 0.01% CaCl_2_ for lipase, and 2% skim milk for protease. Following incubation, enzymatic activity was visualized according to the procedures described by Proca et al. [53]. The strain’s complete hydrolytic enzymatic profile was subsequently assessed with the API ZYM system (BioMérieux, Montalieu-Vercieu, France) according to the manufacturer’s protocol. For qualitative reporting, the results were interpreted as follows: (−) no activity, (±) borderline activity, and (+) positive activity, by the methodology established by Boiu-Sicuia et al. [28].

#### 2.3.5. Assessment of Cell Surface Properties of *Bacillus velezensis* 24.5

Cell surface properties were evaluated following the procedure of Pompa et al. [7], with slight modifications. Overnight cultures of *Bacillus velezensis* 24.5 were centrifuged (4000 rpm, 5 min), washed twice with 1× PBS, and adjusted to OD_600_ = 0.550 ± 0.010 (A0). Auto-aggregation was assessed by incubating cell suspensions at 37 °C, with optical density at 600 nm (OD_600_) recorded after 2 h, 5 h, and 24 h (Af). Hydrophobicity was assessed by mixing 3 mL of bacterial suspension with 1 mL of hexane (apolar solvent), xylene (apolar, aromatic solvent), or ethyl acetate (monopolar basic solvent), incubating at 37 °C, and measuring the OD_600_ of the aqueous phase (Af) after 2 h. Auto-aggregation (%) and hydrophobicity (%) were calculated using the following formula:

$$Auto-aggregation\;and\;hydrophobicity\;(\%)=\frac{A0-Af}{A0}\times 100$$

Co-aggregation ability was assessed by mixing equal volumes (2 mL) of strain 24.5 (10^8^ cells/mL) and each pathogenic indicator (*Staph. aureus* ATCC 33592 or *E. coli* ATCC 8739; 10^8^ cells/mL) and incubating at 37 °C for 5 h. The OD_600_ of the mixtures (Amix) and the individual suspensions (A24.5, Apat) were measured, and co-aggregation (%) was calculated using the following formula:

$$Co-aggregation\;(\%)=\frac{(A24.5+Apat)-2Amix}{A24.5+Apat}\times 100$$

### 2.4. Safety Assessment

#### 2.4.1. Haemolytic Activity Assay

Haemolytic activity was determined according to the method of Coulibaly et al. [52]. Overnight cultures were spotted onto blood agar plates containing 5% (*w*/*v*) defibrinated sheep blood (Oxoid, Basingstoke, Hampshire, UK) and incubated at 37 °C for 14–48 h. β-haemolysis was indicated by a transparent halo around colonies, α-haemolysis by a greenish halo, and γ-haemolysis by no visible change in the surrounding medium. *Staph. aureus* ATCC 33592 was used as a control for β-haemolytic activity.

#### 2.4.2. Antibiotic Susceptibility Testing

The antimicrobial susceptibility of strain 24.5 was evaluated using the disc diffusion method on Mueller–Hinton agar (MHA) following CLSI guidelines (M100) [54]. An overnight culture grown in tryptic soy broth (TSB) was adjusted to a 0.5 McFarland turbidity standard (approximately 10^8^ CFU/mL) using sterile saline. The suspension was evenly spread over the surface of MHA plates (90 mm diameter) under aseptic conditions.

Standardized antibiotic discs (BioAnalyse, Ankara, Turkey) were applied, comprising 24 antibiotics from different classes: β-lactams—amoxicillin/clavulanic acid (AMC20/10), ampicillin (AM10), penicillin (P2U), cefalexin (CL30), ceftriaxone (CRO30), cefuroxime sodium (CXM30); aminoglycosides—amikacin (AK10), gentamicin (CN30), kanamycin (K30), streptomycin (S10); polypeptides—bacitracin (B10U), colistin (CT10); fluoroquinolones/quinolones—ciprofloxacin (CIP1), nalidixic acid (NA30), norfloxacin (NOR30); phenicols—chloramphenicol (C30); macrolides—erythromycin (E10); lincosamides—lincomycin (L10); nitrofurans—nitrofurantoin (F300); tetracyclines—oxytetracycline (T30), tetracycline (TE30); folate pathway inhibitors—trimethoprim/sulfamethoxazole (SXT25); glycopeptides—vancomycin (VA10); and antifungals—fluconazole (FLU10).

Four discs were aseptically placed on each plate, maintaining an inter-disc distance of approximately 3 cm and a minimum distance of 1.5 cm from the plate edge. Plates were incubated at 37 °C for 18–24 h, and inhibition zone diameters were measured to the nearest millimeter using a ruler. The strain was classified as susceptible (S) when the inhibition zone measured ≥ 20 mm, intermediate (I) when between 10 mm and <20 mm, and resistant (R) when ≤10 mm.

### 2.5. Statistical Analysis

Experiments were conducted in triplicate (*n* = 3), and data are presented as mean ± standard deviation (SD). Differences were analyzed using one-way ANOVA in IBM SPSS Statistics software, version 30 (IBM Corp., Armonk, NY, USA), and values of *p* < 0.05 were considered statistically significant.

## 3. Results

### 3.1. Molecular Characterization of the 24.5 Strain

#### 3.1.1. Molecular Identification

**Figure 1:**
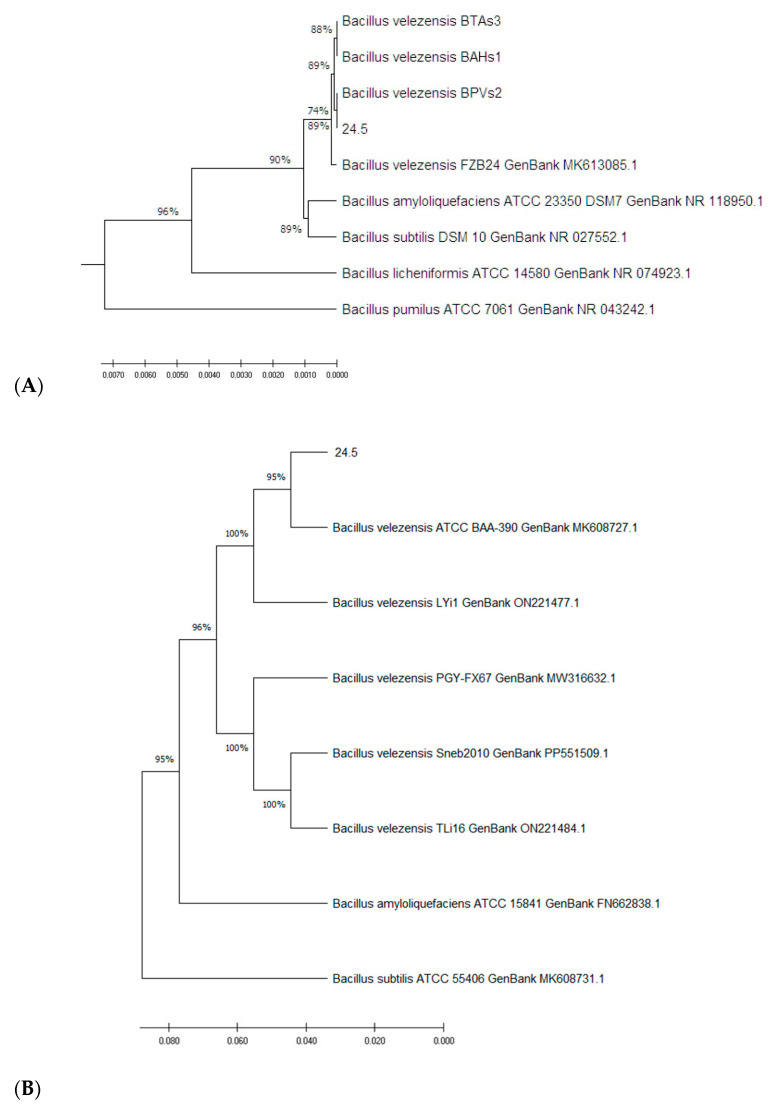
Phylogenetic trees based on 16S rRNA (**A**) and *gyrA* (**B**) gene sequences constructed using MEGA X with ClustalW alignment and the neighbor-joining method. Bootstrap values (1000 replicates) are shown at branch nodes.

Strain 24.5 used in this study was identified at the species level based on 16S rRNA and *gyrA* gene sequence similarity with related bacterial strains in the NCBI database. The 16S rRNA sequence of strain 24.5 showed 99.93% similarity to multiple *Bacillus velezensis* sequences and was deposited in the NCBI database under accession number PX381205. To improve taxonomic resolution, the *gyrA* gene, which encodes DNA gyrase subunit A, was partially sequenced. This *gyrA* fragment showed 99.38–99.90% identity and high query coverage with 88 *B. velezensis* sequences in the NCBI database, supporting its classification as *B. velezensis*. The 16S rRNA and *gyrA* sequences were aligned with those from reference strains using ClustalW in MEGA X software (version 10.1.8), and a phylogenetic tree was constructed based on this alignment (Figure 1A,B).

#### 3.1.2. PCR Detection of Biosynthetic Genes for Secondary Metabolites

Genomic DNA from *Bacillus velezensis* 24.5 was analyzed by singleplex PCR using twelve gene-specific primer pairs targeting reported bioactive metabolite-encoding genes (Table 1). PCR screening revealed that strain 24.5 carries a diverse set of biosynthetic genes responsible for the production of bioactive secondary metabolites. Among the lipopeptide clusters, genes encoding fengycin (*fen*), iturins (*ituD*, *ituA*), surfactins (*srf/lch*, *srfA*), bacillomycin (*bmyA*), and mycosubtilin (*myc*) were detected, whereas *ituC* was absent. All targeted polyketide biosynthetic genes—*dfnA* (difficidin), *mnlA* (macrolactin), and *baeA* (bacillaene)—were detected. Additionally, the dipeptide *bacA/B* gene associated with bacilysin synthesis was identified (Table 2).

### 3.2. Probiotic Patterns of Strain 24.5

#### 3.2.1. Antimicrobial Pattern

Strain 24.5 demonstrated strong antimicrobial activity against several clinically and veterinary relevant Gram-positive pathogens (Table 3). Notably, *Rhodococcus* equi, a primary cause of pneumonia in foals and an opportunistic pathogen in immunocompromised humans, was highly susceptible, as were *Staphylococcus aureus* (including also MRSA) and *Staph. epidermidis* (including MRSE), which are major nosocomial agents. Significant inhibition was also observed for *Enterococcus faecium* and *Listeria monocytogenes*, both important foodborne and zoonotic pathogens. Activity against Gram-negative bacteria was limited, with *Proteus vulgaris* being moderately affected and *Salmonella* spp., *Ps. aeruginosa*, and *S. marcescens* also showing low susceptibility. No inhibition against *Escherichia coli* was detected. Strong antifungal effects against *Candida albicans* and *C. parapsilosis* further emphasize the potential of strain 24.5 for controlling clinically important yeasts. These findings highlight the strain’s potential for future applications in both clinical and veterinary settings, consistent with *Bacillus*-derived antimicrobial lipopeptides.

#### 3.2.2. Resistance to Simulated Gastrointestinal Conditions

**Figure 2:**
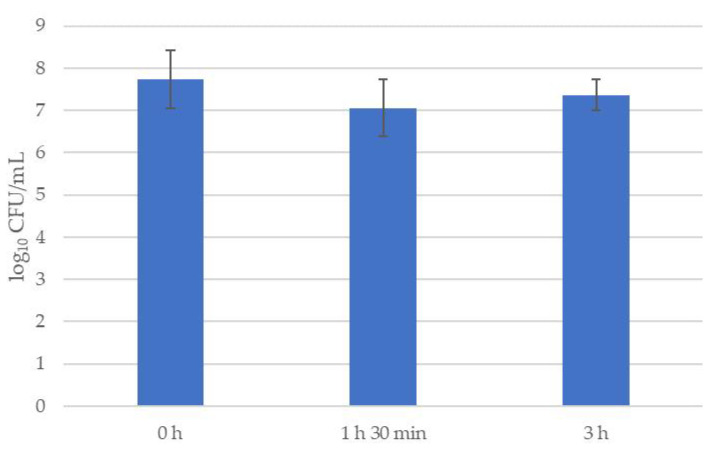
Viability (log_10_ CFU/mL) of *B. velezensis* strain 24.5 under simulated gastric conditions.

The survival of *B. velezensis* strain 24.5 under simulated gastrointestinal conditions is presented in Figure 2 and Table 4.

Cell viability of *B. velezensis* strain 24.5 remained stable under simulated gastric conditions throughout the 3 h incubation. Viable counts decreased from 7.8 log_10_ CFU/mL to approximately 7.0 log_10_ CFU/mL at 1.5 h, followed by a slight increase to ~7.4 log_10_ CFU/mL at 3 h. This stability suggests that strain 24.5 possesses resilience under the tested conditions, a desirable characteristic for maintaining functional viability in probiotic applications (Figure 2).

Strain 24.5 showed high tolerance to bile salts, with viability consistently above 100% across all tested concentrations (0.3–2%) after 4 h under simulated intestinal conditions (Table 4). Although a gradual decrease in viable counts was observed with increasing bile salt concentration, the reductions were minimal, even at 2% bile salts (7.61 log_10_ CFU/mL; 104.40% viability).

These findings suggest that *B. velezensis* 24.5 possesses robust physiological traits for gastrointestinal survival, indicating its potential as a probiotic candidate capable of maintaining viability during gastric passage and subsequent exposure to bile salts.

#### 3.2.3. Antioxidant Activity

As shown in Table 5, *Bacillus velezensis* strain 24.5 demonstrated substantial antioxidant potential, with intact cells exhibiting significantly higher DPPH radical-scavenging activity than the cell-free extract (*p* < 0.05).

#### 3.2.4. Enzymatic Profile of Strain 24.5

Enzymatic screening of the *B. velezensis* strain 24.5 through plate assays showed clear hydrolytic activity for amylase, cellulase, and protease. The API ZYM analysis confirmed the production of esterase, esterase lipase, and naphthol-AS-BI-phosphohydrolase, and indicated borderline alkaline phosphatase activity. However, neither method detected lipase activity (Table 6).

#### 3.2.5. Cell Surface Properties of Halotolerant Strain 24.5

Strain 24.5 exhibited a clear, time-dependent increase in auto-aggregation, rising from 18.60 ± 0.31% at 2 h to 84.42 ± 0.27% at 24 h. The 24 h value was significantly higher (*p* < 0.05) than those at earlier time points, indicating a strong capacity for cell–cell adhesion (Table 7). Hydrophobicity assays showed the highest affinity toward hexane (71.62 ± 0.32%), followed by ethyl acetate (53.89 ± 0.15%), and xylene (34.34 ± 0.13%). These differences were statistically significant (*p* < 0.05), suggesting a predominantly hydrophobic cell surface (Table 7). Strain 24.5 displayed effective co-aggregation with *S. aureus* (39.42 ± 0.45%) and moderate co-aggregation with *E. coli* (28.25 ± 0.29%) (*p* < 0.05).

### 3.3. Safety Traits of Strain 24.5

#### 3.3.1. Haemolytic Activity

Strain 24.5 showed no evidence of haemolysis, consistent with γ-hemolysis, as indicated by the absence of any visible change in the blood agar medium.

#### 3.3.2. Antibiotic Susceptibility Profile

According to CLSI interpretive criteria, *B. velezensis* strain 24.5 was susceptible to 9 antibiotics, exhibited intermediate responses to 13 antibiotics, and was resistant to only two antibiotics (Table 8). The resistance was limited to bacitracin and fluconazole, neither of which is considered critical for human medicine.

## 4. Discussion

While probiotic research has traditionally focused on lactic acid bacteria (LAB) such as *Lactobacillus* and *Bifidobacterium* [3,4,5,6], their practical application can be constrained by susceptibility to adverse environmental conditions and reduced viability during processing or gastrointestinal transit [16,17]. In contrast, *Bacillus* species have gained attention as next-generation probiotics due to their spore-forming capacity, metabolic versatility, and resilience under harsh environmental and gastrointestinal conditions. Although recognized with QPS status by the European Food Safety Authority [25], *B. velezensis* strains still require rigorous, strain-specific evaluation to confirm probiotic efficacy and safety. This study evaluated the probiotic and safety traits of strain 24.5, including probiotic traits (antimicrobial activity, gastrointestinal survival, cell surface traits, enzymatic and antioxidant capacities) and safety properties (molecular characterization, hemolytic activity, antibiotic susceptibility). Initially described as *Bacillus amyloliquefaciens* by Proca et al. [29], strain 24.5 was reclassified as *B. velezensis* in this study based on 16S rDNA and *gyrA* gene analyses, which showed ≥99.38% similarity and confirmed its identification as *B. velezensis*. PCR analysis confirmed the presence of 11 out of the 12 targeted biosynthetic genes, including those encoding lipopeptides (fengycin, iturins, surfactins, bacillomycin, and mycosubtilin), polyketides (difficidin, macrolactin, and bacillaene), and the dipeptide bacilysin. These metabolites are well known for their membrane-disruptive, bactericidal, and fungicidal activities [23,55,56], which likely underpin the broad-spectrum antibacterial and anti-*Candida* effects observed in this study. The pronounced inhibition zones against *Staph. aureus*, *Staph. epidermidis* (MRSA and MRSE), *Strep. pyogenes*, and *C. albicans* are consistent with the established antimicrobial spectra of these compounds. Toma et al. [50] demonstrated that seven *Bacillus* endophytes exhibited broad-spectrum activity, inhibiting 19 of 24 tested bacterial strains, including high-priority pathogens such as *B. cereus*, *E. coli, L. monocytogenes*, *S.* Typhimurium, and *Staph. aureus*. Chen et al. [30] reported that *B. velezensis* TS5 also demonstrated vigorous antagonistic activity against *Staph. aureus*, *Salmonella*, enterotoxigenic *Escherichia coli*, and *E. coli*. Proca et al. [29] previously identified strain 24.5 as exhibiting pronounced antifungal activity against a broad spectrum of phytopathogenic fungi, including *Botrytis cinerea*, *Aspergillus* spp. (*A. carbonarius*, *A. niger*, and *A. flavus*), *Penicillium digitatum*, *Fusarium oxysporum*, and *Alternaria alternata*.

The ability of probiotic candidates to maintain viable cell counts during gastrointestinal transit is a key factor in their potential functional effectiveness. Strain 24.5 showed minimal viability loss over a 3 h incubation under simulated gastric conditions. Furthermore, strain 24.5 maintained over 100% viability after 4 h in bile salt concentrations up to 2%, indicating strong tolerance to intestinal-like conditions. Such resilience, comparable to or greater than that of reported probiotic *Bacillus* strains, indicates effective stress-adaptation mechanisms and supports its potential to survive gastrointestinal transit, reinforcing its suitability as a probiotic candidate. Similar findings have been reported for *B. aryabhattai*, *B. velezensis*, and *B. mojavensis*, which also maintained high survival rates under simulated gastrointestinal conditions [57]. In another study, Alizadeh Behbahani et al. [58] reported that several *Bacillus* species, including *B. cereus*, *B. thuringiensis*, *B. subtilis*, and *B. velezensis*, demonstrated exceptional survival in simulated gastric juice, with viability exceeding 90% after 4 h, and achieved remarkable survival rates of over 145% in simulated intestinal fluid. Nwagu et al. [59] reported that *Bacillus cereus* isolated from traditionally fermented African locust bean seeds exhibited notable bile salt resistance, maintaining over 83% viability after 3 h in MRS broth containing 0.4% bile salts.

Antioxidant-producing probiotics have been reported to protect intestinal epithelial cells from oxidative damage, modulate redox balance, and support gut barrier integrity [60]. The high antioxidant activity observed in *B. velezensis* 24.5 (34.68% for intact cells and 18.47% for cell-free extracts) indicates a strong capacity to neutralize free radicals, a trait of particular importance for probiotic applications. The significantly higher DPPH radical-scavenging activity observed in intact cells compared to the cell-free extract may be attributed to multiple structural and enzymatic factors. Components of the bacterial cell wall, such as peptidoglycan, teichoic acids, and surface-bound polysaccharides, can directly interact with and neutralize free radicals, enhancing antioxidant potential [60]. Moreover, intact cells retain intracellular antioxidant enzymes (e.g., superoxide dismutase, catalase) and redox-active metabolites that are either absent or present at lower levels in the cell-free supernatant [60]. Consistent with these results, Khan et al. [61] demonstrated that a peptide purified from the culture broth of *B. velezensis* exhibited marked antioxidant potential, as shown by high activity in DPPH and ABTS radical-scavenging assays as well as ferric- and cupric-reducing power tests. Similarly, Shivangi et al. [62] reported that *Bacillus* spp. isolated from the acidic fermented food *Idli* displayed notable DPPH radical-scavenging activity, ranging from 26–41% for intact cells and 18–33% for cell-free extracts. Furthermore, Alizadeh Behbahani et al. [58] found that *Bacillus* strains recovered from dairy sludge exhibited substantial DPPH and hydroxyl radical-scavenging activities, with values between 23.32% and 43.52%. Overall, these findings highlight *B. velezensis* 24.5 as a promising probiotic candidate with strong antioxidant properties, suitable for incorporation into functional foods aimed at improving host oxidative balance.

The enzymatic profile of *B. velezensis* 24.5 revealed hydrolytic activity for amylase, cellulase, protease, esterase, esterase lipase, and naphthol-AS-BI-phosphohydrolase, with only borderline alkaline phosphatase activity, while lipase activity was absent. Our results are in agreement with Boiu-Sicuia et al. [28] and Proca et al. [29]. Chen et al. [30] reported that *B. velezensis* TS5 exhibits strong potential to produce not only amylase, cellulase, and protease, but also lipase. From a nutritional perspective, the diverse extracellular hydrolytic enzymes produced by *B. velezensis* 24.5 suggest its potential to enhance host digestion and nutrient utilization, while also contributing to competitive exclusion of pathogens by reducing available substrates in the gastrointestinal environment, thereby complementing its antimicrobial and probiotic functions.

The strong 24 h auto-aggregation, high hexane affinity, and significant co-aggregation with pathogens underscore the versatility and probiotic potential of strain 24.5, aligning with previous reports that link these surface properties to gut colonization and pathogen inhibition [63,64].

The absence of haemolytic activity aligns with the European Food Safety Authority (EFSA) safety requirements for probiotic candidates, as γ-hemolysis indicates a lack of red blood cell lysis and minimizes the risk of cytotoxic effects. This trait is considered essential for strains intended for food or feed applications, ensuring they meet Qualified Presumption of Safety (QPS) standards [25].

Probiotic supplementation is often beneficial during or after antibiotic therapy, as antibiotics can disrupt the intestinal microbiota and predispose individuals to disorders such as diarrhea, acute gastroenteritis, or irritable bowel syndrome. *B. velezensis* 24.5 demonstrated a broad susceptibility spectrum across multiple antibiotic classes, supporting its potential safe application as a probiotic strain.

Overall, *B. velezensis* 24.5 emerges as a versatile probiotic candidate, suitable for incorporation into functional feeds or foods and nutraceuticals, as well as for use in adjunct antimicrobial strategies. By combining antimicrobial, antioxidant, cell-surface, and digestive-support properties, the strain 24.5 may serve as a promising candidate to reduce reliance on chemical preservatives and combat multidrug-resistant pathogens.

## 5. Conclusions

*Bacillus velezensis* strain 24.5 demonstrated a robust probiotic profile, with high survival under simulated gastric and bile conditions, broad-spectrum antimicrobial activity, and diverse extracellular enzymatic capabilities. PCR screening showed that *B*. *velezensis* 24.5 possesses eleven of the twelve investigated biosynthetic genes, encompassing those responsible for producing lipopeptides (iturin, fengycin, surfactin), polyketides, and dipeptides, which are presumed to play a central role in its broad antimicrobial profile. The strain also exhibited strong antioxidant activity, absence of hemolytic activity, and a favorable antibiotic susceptibility pattern, meeting essential safety criteria for Qualified Presumption of Safety (QPS) status. These results highlight the promising probiotic, antimicrobial, and antioxidant properties of *Bacillus velezensis* 24.5, but several limitations should be recognized. Although γ-hemolysis and antibiotic susceptibility testing suggest safety, comprehensive validation, including virulence gene screening and acute or subacute toxicity studies, remains necessary. In addition, stability and viability under industrial processing and storage were not assessed. Nonetheless, the use of GC–MS and UHPLC will offer a detailed characterization of the strain’s metabolite profiles. Addressing these aspects with in vivo experiments, genomic analyses, and formulation studies will be crucial to confirm strain 24.5’s suitability for clinical, veterinary, and food applications.

## Figures and Tables

**Table 1:** Oligonucleotide sequences of genes encoding bioactive metabolites.

Secondary Metabolite Groups	Bioactive Metabolites	Genes	Primers	Primer Sequence 5′-3′	Alignment Temperature	References
Lipopeptides	Fengycin	*fen*	Af2 F	GAATAYMTCGGMCGTMTKGA	45 °C	[46]
			Tf1 R	GCTTTWADKGAATSBCCGCC		
	Iturin	*ituD*	ITUD F1	TTGAAYGTCAGYGCSCCTTT	55 °C	[47]
			ITUD R1	TGCGMAAATAATGGSGTCGT		
	Iturin	*ituC*	ITUC F1	CCCCCTCGGTCAAGTGAATA	55 °C	[47]
			ITUC R1	TTGGTTAAGCCCTGATGCTC		
	Iturin A	*ituA*	ITUD1 F ITUD1 R	GATGCGATCTCCTTGGATGT	55 °C	[48]
				ATCGTCATGTGCTGCTTGAG		
	Surfactin	*srf/lch*	As1 F	CGCGGMTACCGVATYGAGC	43 °C	[46]
			Ts2 R	ATBCCTTTBTWDGAATGTCCGCC		
	Surfactin	*srfA*	SrfA F	AGAGCACATTGAGCGTTACAAA	55 °C	[47]
			SrfA R	CAGCATCTCGTTCAACTTTCAC		
	Bacillomycin	*bmyA*	bmyA F	CTCATTGCTGCCGCTCAATC	55 °C	[49]
			bmyA R	CCGAATCTACGAGGGGAACG		
	Mycosubtilin	*myc*	Am1 F	CAKCARGTSAAAATYCGMGG	45 °C	[47]
			Tm1 R	CCDASATCAAARAADTTATC		
Polyketides	Difficidin	*dfnA*	dfnA F	GGATTCAGGAGGGCATACCG	55 °C	[49]
			dfnA R	ATTGATTAAACGCGCCGAGC		
	Macrolactin	*mnlA*	mlnA F	CCGTGATCGGACTGGATGAG	55 °C	[49]
			mlnA R	CATCGCACCTGCCAAATACG		
	Bacillaene	*baeA*	BaeR F	ATGTCAGCTCAGTTTCCGCA	55 °C	[49]
			BaeR R	GATCGCCGTCTTCAATTGCC		
Dipeptide	Bacilysin	*bacA/B* *bac B*	baeA/B F	TGCTCTGTTATAGCGCGGAG	55 °C	[49]
			baeA/B R	GTCATCGTATCCCACCCGTC		

**Table 2:** Detection of biosynthetic genes for secondary metabolites.

Secondary Metabolite Groups	Bioactive Metabolites	Genes	Expected Product Size (bp)	Detected PCR Product
Lipopeptides	Fengycin	*fen*	443, 452	yes *
	Iturin	*ituD*	482	yes *
	Iturin	*ituC*	594	no
	Iturin A	*ituA*	647	yes *
	Surfactin	*srf/lch*	419–431	yes *
	Surfactin	*srfA*	626	yes
	Bacillomycin	*bmyA*	853	yes *
	Mycosubtilin	*myc*	416, 419	yes
Polyketides	Difficidin	*dfnA*	653	yes
	Macrolactin	*mnlA*	668	yes
	Bacillaene	*baeA*	688	yes
Dipeptides	Bacilysin	*bacA/B* *bac B*	910	yes

* Previously reported by Proca et al. [29].

**Table 3:** Growth inhibition of reference microorganisms by *Bacillus velezensis* strain 24.5.

Bacteria Gram-Positive			Bacteria Gram-Negative			Fungi		
Pathogens	Aspect	Inhibition Halo (mm)	Pathogens	Aspect	Inhibition Halo (mm)	*Candida*	Aspect	Inhibition Halo (mm)
*B. cereus* ATCC 11778	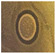	2.00 ± 0.00 ^f^	*E. coli* ATCC 11229	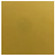	0.00 ± 0.00 ^g^	*C. albicans* ATCC 10231	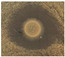	15.33 ± 0.94 ^b^
*Ent. faecium* ATCC 6057	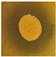	9.00 ± 0.82 ^cd^	*E. coli* ATCC 8739	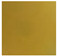	0.00 ± 0.00 ^g^	*C. glabrata* ATCC 2001	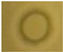	3.00 ± 0.82 ^f^
*Ent. faecalis* ATCC 29212	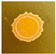	2.33 ± 0.47 ^f^	*Ps. aeruginosa* ATCC 15442	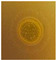	1.00 ± 0.00 ^f^	*C. parapsilopsis* ATCC 20019	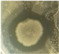	8.33 ± 0.47 ^cd^
*Ent. hirae ATCC 10541*	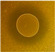	5.33 ± 0.47 ^e^	*Ps. aeruginosa* ATCC 27853	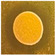	2.00 ± 0.00 ^f^	*C. tropicalis* ATCC 44508	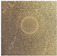	0.00 ± 0.00 ^g^
*L. ivanovii* ATCC 19119	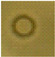	4.00 ± 0.00 ^e^^f^	*Pr. vulgaris* ATCC 13315	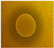	5.67 ± 0.47 ^d^^e^			
*L. monocytogens* ATCC 7644	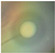	9.00 ± 0.00 ^cd^	*S. enterica* Typhimurium ATCC 14028	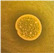	1.67 ± 0.47 ^f^			
*L. innocua* ATCC 33090	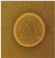	1.00 ± 0.00 ^f^	*S. enterica* Enteritidis ATCC 13076	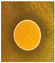	2.67 ± 0.47 ^f^			
*Staph. aureus* ATCC 25923	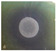	10.67 ± 0.94 ^b^^c^	*S. marcescens* ATCC 14756	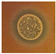	2.33 ± 0.47 ^f^			
*Staph. aureus* ATCC 33592 MRSA	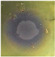	10.67 ± 0.94 ^b^^c^						
*Staph. epidermidis* ATCC 51625	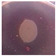	12.33 ± 0.47 ^b^						
*Staph. epidermidis* ATCC 12228	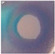	13.00 ± 0.82 ^b^						
*Strep. pyogenes* ATCC 19615	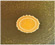	4.67 ± 0.47 ^e^^f^						
*R. equi* ATCC 8939	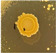	16.67 ± 0.94 ^a^						

Values are reported as the mean ± standard deviation (SD). Different superscript letters within each column indicate significant differences (*p* < 0.05).

**Table 4:** Effect of different bile salt concentrations on the survival of strain 24.5 under simulated intestinal conditions.

Bile Salt Concentrations	Initial Time	Simulated Intestinal Conditions			
		2 h		4 h	
		Log_10_ CFU/mL	% Viability	Log_10_ CFU/mL	% Viability
Control	7.50 ± 0.09	8.64 ± 0.08	115.11 ± 0.93	10.56 ± 0.10	140.70 ± 0.28
0.3%	7.37 ± 0.08	8.15 ± 0.15	110.59 ± 0.94	9.88 ± 0.07	134.02 ± 1.67
0.5%	7.32 ± 0.09	7.38 ± 0.15	100.77 ± 0.92	8.52 ± 0.11	116.39 ± 1.50
1%	7.39 ± 0.02	7.75 ± 0.14	104.97 ± 1.70	8.21 ± 0.02	111.10 ± 0.88
2%	7.29 0.09	7.34 ± 0.07	100.60 ± 0.75	7.61 ± 0.05	104.40 ± 1.60

**Table 5:** Antioxidant activity of *Bacillus velezensis* strain 24.5.

Strain	Sample Type	Antioxidant Activity (% ± SD)
24.5	Intact cells	34.68 ± 1.33 ^a^
	Cell-free extract	18.47 ± 1.69 ^b^

Values represent mean ± SD of triplicate measurements. Different superscript letters within a column indicate significant differences (*p* < 0.05).

**Table 6:** Hydrolytic enzyme activities of *Bacillus velezensis* strain 24.5.

Strain	Plate Screening				Api Zym Kit			
	Amylase	Cellulase	Lipase	Protease	Alkaline Phosphatase 2	Esterase (C4) 3	Esterase Lipase (C8) 4	Naphthol-AS-BI-Phosphohydrolase 11
24.5	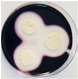	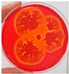	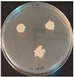	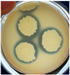	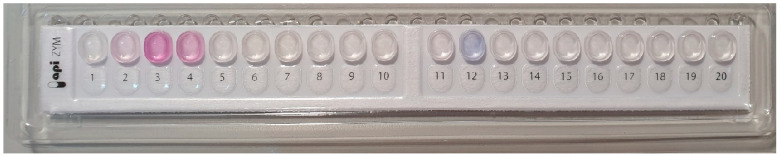			
	+	+	-	+	±	+	+	+

- no activity, ± borderline activity, and + positive activity.

**Table 7:** Auto-aggregation, Hydrophobicity, and Co-aggregation Properties of Halotolerant Strain 24.5.

Strain	Auto-Aggregation (%)			Hydrophobicity (%)			Co-Aggregation (%)	
	2 h	5 h	24 h	Hexane	Xylene	Ethyl Acetate	*S. aureus*	*E. coli*
24.5	18.60 ± 0.31 ^c^	27.97 ± 0.12 ^b^	84.42 ± 0.27 ^a^	71.62 ±0.32 ^a^	34.34 ± 0.13 ^c^	53.89 ± 0.15 ^b^	39.42 ± 0.45 ^a^	28.25 ± 0.29 ^b^

Values are means ± standard deviations; different superscript letters within a column indicate significant differences, *p* < 0.05.

**Table 8:** Interpretation of Antimicrobial Resistance Patterns in Strain 24.5.

Drug Class	Antibiotic (Code)	Interpretation *
β-lactams	Amoxicillin/clavulanic acid (AMC20/10)	I
	Ampicillin (AM10)	I
	Cefalexin (CL30)	S
	Ceftriaxone (CRO30)	S
	Cefuroxime sodium (CXM30)	I
	Penicillin (P2)	I
Aminoglycosides	Amikacin (AK10)	S
	Gentamicin (CN30)	S
	Kanamycin (K30)	S
	Streptomycin (S10)	I
Polypeptides	Bacitracin (B10)	R
	Colistin (CT10)	I
Fluoroquinolones/Quinolones	Ciprofloxacin (CIP1)	S
	Nalidixic acid (NA30)	I
	Norfloxacin (NOR30)	S
Phenicols	Chloramphenicol (C30)	S
Macrolides	Erythromycin (E10)	I
Lincosamides	Lincomycin (L10)	I
Nitrofurans	Nitrofurantoin (F300)	I
Tetracyclines	Oxytetracycline (T30)	I
	Tetracycline (TE30)	I
Folate pathway inhibitors	Trimethoprim/sulphamethoxazole (SXT25)	S
Glycopeptides	Vancomycin (VA10)	I
Antifungals	Fluconazole (FLU10)	R

* Interpretation criteria: S (Susceptible) ≥ 20 mm, I (Intermediate) = 10–19 mm, R (Resistant) ≤ 10 mm.

## Data Availability

The original contributions presented in this study are included in the article. Further inquiries can be directed to the corresponding author.
